# Is procalcitonin a valuable marker for identification of postoperative complications after coronary artery bypass graft surgery with cardiopulmonary bypass?

**DOI:** 10.1186/cc14352

**Published:** 2015-03-16

**Authors:** A Baysal, M Dogukan, H Toman

**Affiliations:** 1 Kartal Kouyolu Research and Training Hospital, Istanbul, Turkey; 2 Adiyaman University Research and Training Hospital, Adiyaman, Turkey; 3 Canakkale 18 Mart University Hospital, Canakkale, Turkey

## Introduction

The aim of our study was to investigate the value of C-reactive protein (CRP) and procalcitonin (PCT) in identification of the systemic inflammatory response syndrome (SIRS) and other complications in the early postoperative period after cardiac surgery with cardiopulmonary bypass (CPB) [1].

## Methods

In 93 patients undergoing coronary artery bypass graft surgery with CPB, after Ethical Committee approval in a prospective study, serum PCT and CRP values were collected before operation and daily until postoperative day 5. All patients were divided *post hoc *into patients with SIRS (*n *= 42) and patients without SIRS (*n *= 51). Student's *t *test, the Mann-Whitney *U *test and receiver operating characteristic (ROC) curves were used.

## Results

The comparison of serum CRP values in patients with or without SIRS on postoperative day 1 until postoperative day 5 demonstrated an increase in both groups without significant differences (*P >*0.05). The PCT levels increased more significantly in SIRS patients (5.78 ± 3.21 ng/ml vs. 1.23 ± 0.31 ng/ml) compared with patients without SIRS (*P *= 0.0001) on postoperative day 1. In patients with postoperative complications (21/93, 22%) (circulatory failure = 10, pneumonia = 2, respiratory insufficiency = 9, sepsis = 0), PCT levels remained elevated until postoperative day 5 (6.11 ± 2.87 ng/ml) but diminished in patients with SIRS (0.96 ± 0.23 ng/ml) (*P *< 0.0001). A PCT threshold value of 2.79 ng/ml was able to discriminate between postoperative complications in patients with or without SIRS with a sensitivity of 82.5% and a specificity of 70% (area under the curve: 0.76 ± 0.05; *P *< 0.01) on postoperative day 1.

## Conclusion

After cardiac surgery with CPB, PCT values increased significantly in the SIRS group of patients compared with patients without SIRS on postoperative day 1 and remained elevated until postoperative day 5. In the early postoperative period, early rise of PCT values may help to discriminate the development of postoperative complications in patients with or without SIRS.
